# Fragility Fracture Prevention—Implementing a Fracture Liaison Service in a High Volume Orthopedic Hospital

**DOI:** 10.3390/ijerph16244902

**Published:** 2019-12-04

**Authors:** Federico Pennestrì, Sabrina Corbetta, Vittoria Favero, Giuseppe Banfi

**Affiliations:** 1 IRCCS Orthopedic Institute Galeazzi, Scientific Direction, 20161 Milan, Italy; banfi.giuseppe@hsr.it; 2 IRCCS Orthopedic Institute Galeazzi, Endocrinology and Diabetology Service, 20161 Milan, Italy; sabrina.corbetta@unimi.it (S.C.); vittoria.favero@unimi.it (V.F.); 3 Department of Biomedical, Surgical and Odontoiatric Services, University of Milan, 20122 Milan, Italy; 4 Università Vita-Salute San Raffaele, Scientific Direction, 20132 Milan, Italy

**Keywords:** fracture liaison service, value-based healthcare, osteoporosis prevention, capture the fracture, fragility fracture, endocrinology, sustainability

## Abstract

Fragility fractures pose a serious threat to patient health, quality of life, and healthcare sustainability. In order to reduce their clinical, social, and economic burden, a Fracture Liaison Service (FLS) was introduced in a high volume orthopedic hospital in 2017. The purpose of this retrospective observational study is to describe the FLS protocol, introduce its preliminary outcomes, and provide an early evaluation in light of international guidelines and recommendations. All the performances suggested by the International Osteoporosis Foundation (IOF) are provided under the same institution by which a patient is admitted for surgery. Clinical indicators from patient history and administrative indicators from the hospital database have been used to estimate the spread of fragility fracture prevention and the degree of patient compliance to these programs. The research included 403 patients. Although, almost 1/3 were admitted for the second fragility fracture, only half received anti-osteoporotic treatment before it. The degree of prevention was even lower in the case of patients admitted for the first fragility fracture. The risk of being affected by a secondary fracture was seven times higher when patients did not attend any follow-up or diagnostic exam. In order to identify the main determinants of compliance with FLS and perform a cost-effectiveness analysis on a larger sample, it is fundamental to integrate data from different providers.

## 1. Introduction

Osteoporosis is a bone disease characterized by loss of density and deterioration. When bone resilience is compromised, the patient is exposed to an increased risk of fragility fracture, occurring from low-impact mechanical forces which would normally be tolerated [1].

According to the World Health Organization (WHO), low impact is equal to a fall from standing height or less. Since the patient is often asymptomatic before the fracture occurs, osteoporosis is referred to as a “silent disease.” The annual incidence of osteoporosis has been estimated at 9 million fractures worldwide, which is likely to increase as a consequence of aging [2,3]. In the United States, 10.2 million people are affected by osteoporosis and 43.4 million by low bone mass, equaling a quarter of the whole adult population [4]. The impact is similar in Canada, Europe, and Asia [5,6,7]. In Italy, 90.000 hip fractures affect 50-years old patients each year [8].

On a global scale, the level of disability induced by osteoporotic fractures has been estimated to be 0.832 million and 1.53 million Disability-Adjusted Life-Years (DALYs) for men, and women, respectively [3]. Osteoporotic fragility fractures cause substantial pain and severe disability, leading to a reduction in life expectancy and quality of life. Hip fractures, in particular, are associated with a significant social and healthcare burden. About 24% of patients ≥ 50 years old die within one year following a hip fracture [9], while nearly 50% suffer long-term disability or require long-term nursing home care (25%) [10].

In addition to this burden, the economic costs of fragility fractures are also substantial. In the UK, over 300.000 patients present to hospitals with fragility fractures each year. The social and medical costs of fragility fractures to the UK healthcare economy were estimated at £1.8 billion in 2000, with a potential increase to £2.2 billion. Hip fractures are the most significant type of fragility fracture because of the human impact and the need for long-term institutional care, and associated high medical costs [6]. In Italy, the direct hospitalization costs for the treatment of patients with fragility fractures over the age of 65 were almost 400 € million in 2002, with a 15% increase in the following three years [11]. The socio-economic burden of fragility fractures is not unique to Europe, but affects all countries with an aging population, such as Australia [12], Canada [13], China [14], and Taiwan [7]. On average, patients suffering from osteoporotic fractures increase their risk of exposure to a second fracture by 86%. When the first fracture affects the vertebrae or the hip, the percentage reaches 200%, and 300%, respectively [4]. Despite the well-known benefits of preventive bone health assessment [15] and osteoporotic drugs, most people presenting with a fragility fracture are neither assessed for osteoporosis, nor appropriately managed to prevent further fractures, irrespective of different countries and health care systems [16].

In 2013, the International Osteoporosis Foundation (IOF) published a landmark paper to increase global secondary prevention strategies, recommending the implementation of Fracture Liaison Services (FLS) whenever possible [17]. FLS is a multidisciplinary approach in reducing secondary fractures by identifying patients at risk that have been admitted to surgery, and providing easy and coordinated access to osteoporosis prevention and care. The IOF created a Best Practice framework to standardize and support its spread (Capture the Fracture), introducing a FLS algorithm which can be adapted to the organizational needs and resources of the local provider, healthcare system, and workforce composition [18]. With a considerable increase in protocols, which have been recently published in the literature, original evidence from empirical data is still recommended by systematic and narrative reviews [6,7,19].

The purpose of this retrospective observational study is to support this request by describing the FLS protocol adopted by a high volume orthopedic hospital (Istituto di Ricovero e Cura a Carattere Scientifico Orthopaedic Institute Galeazzi, Milan, Italy), after 26 months of implementation (1 April 2017–31 May 2019); introducing its preliminary outcomes, and providing an early evaluation in light of international guidelines and recommendations.

## 2. Materials and Methods

IRCCS Galeazzi Orthopaedic Institute is the most appropriate hospital in Italy for taking charge of a femur fracture [20,21], and it was recently recognized internationally among hospitals with the best practice in this area [22]. The protocol described here is based on the cooperation between Traumatology and Endocrinology Units, with multiple aims of:

1. *Identifying* patients at risk of osteoporosis-related secondary fracture, both of the upper limb (humerus) and lower limb (femur).
2. *Investigating* biochemical indicators of bone metabolism associated to Bone Mass Density (BMD), to evaluate the degree of osteoporosis-fracture risk to which the patient is exposed, through specific diagnostic exams (Vertebral and Femoral Dual-Energy X-ray Absorptiometry: DXA; and/or Toracolumbar Spine X-ray: TSRx), and eventually.
3. *Initiating* multidisciplinary treatment under the same provider to which the patient was admitted for surgery.

Patients admitted to the Traumatology Unit were evaluated by FLS when the risk of a further fragility fracture was identified by the surgeon or his team. Femur and humerus fractures were only included in the start-up period under consideration, because generally, unlike fractures of the wrist and vertebrae, patients need hospitalization in the Traumatology Unit to recover from surgery. Subsequently, specific osteoporosis assessment and subsequent cycles of care were provided under the coordination of the Endocrinology Unit, composed, in turn, by two endocrinology and one rheumatology outpatient units. Fractures were identified, respectively, by international diagnostic codes ICD-9 812.0-812.59 (humerus) and 820 (femur). All the patients at risk were included in the program, except those affected by postoperative cognitive dysfunction, dementia, or borderline, as the risk of poor compliance with the service was considered too high. Since all the clinical procedures performed were ISO-9000 certified, and because the study had no experimental design, and ethical approval and informed consent were not required.

**Figure 1:**
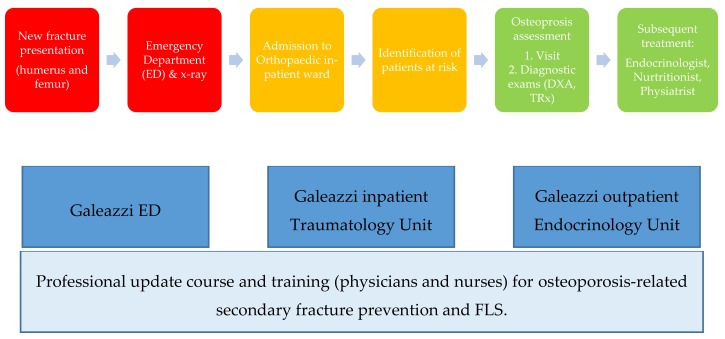
Istituto di Ricovero e Cura a Carattere Scientifico Galeazzi Capture the Fracture protocol.

The protocol adopted by IRCCS Galeazzi is represented in Figure 1.

Clinical and administrative indicators were, respectively, collected from the patient histories and the hospital internal database, and categorized according to the type of evidence they support. Clinical indicators measure the degree of spread, knowledge, and actual implementation of osteoporosis prevention services. Therefore, they provide evidence in support of epidemiology and healthcare policy. Administrative indicators estimate the degree of patient compliance with the service, which is, in turn, a potential indicator of the service effectiveness. Therefore, they may be helpful to improve clinical outcomes and healthcare policy. A methodological overview is summarized in Table 1.

## 3. Results

Over the 26 months of evaluation, 1278 patients were admitted to the Traumatology Unit for proximal fracture of the femur (n = 923) and the humerus (n = 355). Of these patients, 407 were enrolled in FLS (31.8%), of which 4 were not identified in the hospital database. Therefore, 403 patients were included in the research. Among them, mean age was 77.6 years and median age was 79 years.

Clinical and administrative indicators and outcomes are, respectively, reported in Table 2 and Table 3.

## 4. Discussion

### 4.1. Benefits of FLS as Reported in Literature

A systematic review identified four models of osteoporotic fracture prevention, which vary according to the amount of performances provided among the same institution, and the degree of coordination they are able to establish [23]. Models and combinations are represented in Table 4.

According to the same review, fully coordinated, intensive models of care are more effective in improving patient outcomes than models based on patient education only: The more coordination between care-givers and providers, the more clinical and economic benefits. Within a few years and many FLS protocols implemented later, these findings were supported by several reviews and empirical studies. With regard to the clinical benefits, a before-after observational study found significant increases in BMD testing, treatment initiation, adherence, reductions in secondary fractures, and mortality when an integrated FLS program was implemented; no correlations between compliance and age, sex, history of fracture and ongoing drug treatments at admission were found [24]. Not only did FLS yield higher rates of diagnosis and treatment in comparison with other programs, it also improved accessibility to the post-fracture pathway of care, supporting a patient-centered healthcare approach [4]. With regard to the most common fracture sites, the benefits of FLS programs were more significant in non-vertebral (i.e., femur and humerus) than in vertebral fractures [25], probably because there were more therapeutic and diagnostic procedures requested by the latter [26,27]. With regard to the economic benefits, the financial value of FLS relies on the prevention of a high human and monetary burden. A study conducted on UK tariffs in 2004 underlined the cost-effectiveness of secondary fracture prevention [28]. In the event of hip fracture, the price of a DXA scan followed by osteoporosis education (i.e., life-style advice) and/or pharmacological treatment (i.e., bisphosphonates intake) ranged from £23 to £335, which was significantly cheaper than surgical treatment, which ranged in turn from £5000 to £12,000, depending on the inclusion or not of residential rehabilitation support. Although, 15 years have passed and the rates may have been updated, the difference remains significant, not to mention the social burden expressed by disability and the subsequent need for formal or informal assistance [12]. Another study based in the UK found that FLS implementation over a 5-year period saved £290,708 in National Health Service (NHS) acute services, community services, and local authority social costs, as opposed to an additional £234,181 in revenue, covering drug treatment for the same period [29]. From a public health perspective, FLS is, therefore, expected to generate long-term health and economic benefits, both, for the patients and the community, thereby supporting healthcare continuity and culture [26], which is a key-determinant of healthcare value [30,31,32,33].

### 4.2. FLS Challenges and Room for Improvement as Reported in Literature

According to a systematic literature review of 53 high quality studies, the degree of patient compliance with FLS varies significantly with the type of program, depending on several factors, such as the presence and qualification of the coordinator, the type of healthcare system, the amount of financial resources invested, and the quality of education provided [34]. The fracture location may also influence the management of osteoporotic fractures, potentially reflecting clinician bias: BMD testing was performed significantly more in association with humerus fracture (85% of patients) than with hip fracture (21% of patients), improving as a consequence the prevention of the former. More BMD testing was performed in association with forearm factures, rather than with spinal or humerus fractures; and more pharmacological treatment was performed following forearm fractures rather than following spinal fractures. According to the same review, these data suggest the rate of BMD assessment after major bone fracture was lower than following minor bone fracture [34].

Despite that, women are more likely to suffer from a fragility fracture in their lifetime (50% versus 20% for men) [25], correlations between sex and FLS initiation, intensity, and compliance are also controversial [34,35]. Contrary to the hypothesis of an inherent gender bias in the management of osteoporotic fractures [34], Ruggiero et al. found a 1-year adherence to complete pharmacological treatment to be independent of age, sex, history of fracture, and ongoing drug treatment declared during anamnesis at admission [36].

The same review found no direct comparisons about FLS effectiveness in different settings, despite the indirect comparisons of limited data, which show interesting information in support of further studies:

- Higher rates of BMD testing are performed in hospitals rather than in outpatient clinics.
- Higher rates of BMD testing are performed in outpatient clinics rather than in communities, with a high variation according to the program.

However, community programs have also shown significant improvement and accuracy in BMD testing and osteoporosis medication if central coordination was provided. However the algorithm was implemented, and substantial connections between different settings, healthcare cycle phases, and care-givers are key-factors that determine FLS effectiveness. For instance, managed care providers generate cost-effective coordination by bridging the gap between acute hospital and community-based care [37,38,39,40]. This is even more noteworthy in cases of fragile or chronic patients affected by multiple comorbidities, to which an effective FLS program should offer flexible and coordinated cycle of care in an outpatient setting, and extended education and involvement of family members.

### 4.3. Implications for Galeazzi Hospital Protocol

The protocol adopted by Galeazzi is a “Triple 1” (identifying, investigating, initiating), highly integrated model of prevention, in which all performances suggested by the IOF framework are provided under the same institution (or network) where the patient was admitted to surgery. Most of the patients evaluated by FLS are chronic and fragile elderly who take more than three drugs regularly (50.3%), who are often affected by multiple morbidities (30%), which adds significant value to osteoporosis care provision in an outpatient setting. Family education and involvement is also contextually provided.

More than 1/3 (39.4%) of the patients evaluated by FLS (which are, in turn, nearly 1/3 of all the patients admitted for the relevant types of surgery in the same Traumatology Unit) had a history of fragility fracture, despite less than half (47.7%) having received anti-osteoporotic pharmacological treatment after the first trauma. In cases of first fragility fracture, the percentage of those who regularly consumed supplementations of vitamin-D was even lower (29.3%). More prevention of fragility fractures is, therefore, a key point to be introduced in the healthcare policy agenda, both, for primary and secondary care.

Nearly 1/3 of the patients identified at risk were compliant with the entire cycle of osteoporosis care, among which only 1 (0.24%) was still affected by a fragility fracture within one year. The risk of being affected by a secondary fragility fracture was approximately seven times higher when patients did not attend any visit or exam at all (1.73%). The percentage of patients who underwent follow-up diagnostic examinations at the admitting hospital is low (DXA: 1.98%, and DXA plus TRx: 0.49%). Rather than indicating poor effectiveness on part of the service, this data is likely to indicate that patients, once discharged, prefer to attend the full cycle of care as close as possible to their home, even more if they are elderly and frail.

Though, the study design was not meant to compare the rate of secondary fragility fracture before, and after, the implementation of FLS, we could detect a trend in reduction of the number of patients admitted to Traumatology Unit for secondary trauma within one year from the last fracture, comparing admissions before and after the institution of FLS (1 March 2015–31 March 2017; 1 April 2017–31 May 2019) (Table 5).

Before FLS was implemented, fragility fractures were not explicitly reported. Therefore, the rate of secondary fracture was very low as a proportion of all fractures. Once FLS was implemented, all fractures were classified as fragility or not during the identification phase of the algorithm. Thus the increase in secondary fracture is confounded by the classification of fractures according to mechanism of injury—low impact versus not low impact.

### 4.4. Limitations

This study has a number of limitations. On the clinical level, the population included in the program was limited. Fractures other than femur and humerus were excluded. Frail patients affected by cognitive disfunction, dementia, or borderline cases were also excluded. On the administrative level, it was not possible to verify whether the patients, once discharged, underwent the cycle of care at another provider. On the methodological level, it was not possible to perform a clear comparison of the prevalence of secondary fragility fractures before, and after, the implementation of FLS.

Most of these limitations are due to the early stage of service implementation, such as the limited population size, the limited type of fractures, and the exclusion of frail patients. On this regard, the greater number of staff hired in the program, the more the program can gradually include more patients and fractures, for instance, by including the type of fractures that do not require hospitalization in the Traumatology Unit, or by employing dedicated personnel to support people affected by mental conditions which may hinder appropriate compliance. This study could not identify correlations between patient characteristics, care-giver qualification and compliance to the service, nor effectiveness, which is a potentially interesting variable to be investigated by further studies on more patients and procedures. To the best of our knowledge, there is just one Italian study designed as a before-after observational study on the implementation of a FLS, which is still based on internal hospital data and was conducted on a smaller number of patients [24].

Limited data availability underlines the need for prospective and before-after observational studies integrating public or multicenter data. Integrating internal data with external data, and having larger resources available (whether institutional networks or public funded databases) would be a significant step forward in order to firstly, understand how many patients that are admitted, identified, and assessed in Galeazzi may continue their treatment elsewhere, as opposed to interrupting the treatment; secondly, perform a cost-effectiveness analysis on a larger population. Future challenges include how best to measure the success of services in imparting a reduction in fractures at a local population level, as well as how to detect those patients with unmet needs who do not uniformly present to health care services [39]. The hospital commitment to evaluate patient outcomes and experiences by means of an electronic registry has already been started [40].

## 5. Conclusions

FLS is not a quick fix [6]. However, most of its challenges are likely to be overcome by gradual experimentation and assessment, which the present research aims to support. The ideal approach to secondary fracture prevention is to implement a Type-A model of care supported by an integrated electronic health network, supervised by clinical and/or logistical coordinators, with reference to a dedicated database in order to quantify performances [18,41].

From year 2006, Galeazzi has been a member of the International Society of Orthopedic Centers (ISOC), whose aim is to share innovation and best practices among the premier specialty orthopaedic institutions in the world [42]. When multicenter, regional, and/or national data is available from integrated informative systems, it will be possible to validate the benefits of FLS on a higher number of patients, extending the potential benefits to other patients and diseases. The burden of deadly chronic morbidities, such as diabetes and cardiovascular disease may, in turn, be significantly reduced by providing effective patient identification, timely investigation, and appropriate initiation of treatment. Galeazzi’s protocol is compliant with most of the international recommendations introduced in the manuscript. Collaboration between epidemiologists, policy makers, clinics, and also sociologists may offer further room for improvement on a regional and national scale, which is likely to generate important benefits to the whole society. The road to healthcare value is not paved only by financial constraints and merely cost-reduction, but also from a wiser and longer-term allocation of resources.

## Figures and Tables

**Table 1:** Methodological overview.

Database	Indicators	Outcome	Relevance	Potential Biases and Limitations
1. Clinical (medical history of patients enrolled to FLS)	1.1 Regular intake of vitamin D before primary fracture, reported in medical history.	1.1.1 Absolute number of patients. 1.1.2 Percentage out of FLs patients.	To estimate the spread, knowledge and actual implementation of pharmacological and nutritional prevention of osteoporosis-related primary fractures.	Estimations are limited to the experimental sample size.
	1.2 Previous fragility fracture, regardless of the bone involved, reported in medical history.	1.2.1 Absolute number of patients. 1.2.2 Percentage out of FLs patients.	To estimate the general incidence of secondary fractures.	
	1.3 Pharmacological treatment between primary and secondary fracture (among patients reporting previous fragility fractures, 1.2).	1.3.1 Absolute number of patients. 1.3.2 Percentage out of FLs patients.	To estimate the spread, knowledge and actual implementation of pharmacological prevention of osteoporosis-related secondary fractures.	
	1.4 Regular consumption of more than three drugs regardless to osteoporotic prevention.	1.4.1 Absolute number of patients. 1.4.2 Percentage out of FLs patients.	To estimate the degree of comorbidity among the population of patients affected by fragility fractures.	
	1.5 Presence of multiple morbidities.	1.5.1 Absolute number of patients. 1.5.2 Percentage out of FLs patients.		
2. Administrative (hospital internal database)	2.1 Return to hospital for full outpatient osteoporosis care (osteoporosis assessment and subsequent treatment).	2.1.1 Absolute number of patients. 2.1.2 Percentage out of FLs patients.	To estimate the degree of patients’ sensitivity towards the risk of a further fragility fracture, and/or the effectiveness of the education provided by care-givers.	Patients may have undergone follow-up (full or partial) at other healthcare providers.
	2.2 No return to hospital for follow-up outpatient osteoporosis visit, but return to hospital for one diagnostic exam (Vertebral and Femoral Dual-Energy X-ray Absorptiometry: DXA).	2.2.1 Absolute number of patients. 2.2.2 Percentage out of FLs patients.		
	2.3 No return to hospital for follow-up outpatient osteoporotic visit, bur return to hospital for both diagnostic exams (DXA, Toracolumbar Spine X-ray: TSRx).	2.3.1 Absolute number of patients. 2.3.2 Percentage out of FLs patients.		
	2.4 No return to hospital for any outpatient osteoporotic treatment but readmitted for another fragility fracture within one year.	2.4.1 Absolute number of patients with relative sites of fracture. 2.4.2 Percentage out of FLs patients.	To estimate the effectiveness of secondary fracture prevention.	
	2.5 Return to hospital for any outpatient osteoporotic treatment but readmitted for another fragility fracture within one year.	2.5.1 Absolute number of patients with relative sites of fracture. 2.5.2 Percentage out of FLs patients.		

**Table 2:** Clinical evidence.

Indicator	Absolute # Patients	% out of FLS Patients (403)
1.1 Vitamin-D therapy before primary fracture.	117	29.3
1.2 Previous fragility fracture.	159	39.4
1.3 Pharmacological treatment between primary or secondary fracture (subgroup of domain 1.2).	76	47.7 (out of 159)
1.4 Patients affected by multiple comorbidities.	121	30
1.5 Patients regularly assuming more than three drugs.	203	50.3

**Table 3:** Administrative evidence.

Indicator	Absolute # Patients	% Out of FLS Patients (403)
2.1 Patients who came back to outpatient osteoporosis care (visit, exam, treatment).	132	32.70%
2.2 Patients who came back for DXA.	8	1.98%
2.3 Patients who came back for DXA and TRx.	2	0.49%
2.4 Patients who did not come back for osteoporosis care readmitted for another suspected fracture within one year.	7 (5 Spine, 1 Rotula, 1 Wrist fracture)	1.73%
2.5 Patients who came back for osteoporosis care but still were readmitted for another fracture within one year.	1 (Wrist fracture)	0.24%

**Table 4:** Four models of secondary fracture prevention (elaborated by the authors) ^1^.

Model	Identifies Patient	Investigates Osteoporosis	Initiates Treatment(s)	± Effective
Type A (3-I)	*Provided* under the same setting (hospital, integrated providers: i.e., managed care) in which primary fracture is treated (i.e., operating surgeon or his/her collaborators).	*Provided* under the same setting in which primary fracture is treated (i.e., surgeon collaborators, other specialists).	*Provided* under the same setting in which the fracture is treated, in a multidisciplinary and coordinated manner (i.e., rheumatologist, endocrinologist, physiatrist, nutritionist).	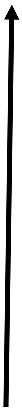
Type B (2-I)	*Provided* as above.	*Provided* as above.	*Referred or recommended* to primary care or other providers	
Type C (1-I)	*Provided* as above.	*Referred or recommended* to primary care or other providers.	*Referred or recommended* as above.	
Type D (0-I)	The patient is *educated* about osteoporosis and given lifestyle advice, included falls prevention, regardless of any relation between fracture and osteoporosis.	No recommendations or referral to primary care or other providers.	No recommendations or referral to primary care or other providers.	

^1^ In green are the treatments provided under the same institution, in red are the treatments referred or recommended to other providers.

**Table 5:** Rate of secondary fractures before and after implementation of Fracture Liaison Service (FLS).

Indicator	Before FLS (2015–2017)	After FLS (2017–2019)
Number of patients admitted to traumatology for the same site of fracture	1220	1278
Number and percentage of patients evaluated by FLS	0 (0%)	403 (31.5%)
Number and percentage of secondary fractures	23 (1.8%) (general fractures)	159 (39.4%) (only fragility fractures)
Number and percentage of secondary fractures within one year	21 (91.3%)	1 (0.62%)
